# Functional Traits and Water Transport Strategies in Lowland Tropical Rainforest Trees

**DOI:** 10.1371/journal.pone.0130799

**Published:** 2015-06-18

**Authors:** Deborah M. G. Apgaua, Françoise Y. Ishida, David Y. P. Tng, Melinda J. Laidlaw, Rubens M. Santos, Rizwana Rumman, Derek Eamus, Joseph A. M. Holtum, Susan G. W. Laurance

**Affiliations:** 1 Departamento de Ciências Florestais, Universidade Federal de Lavras, Lavras, Minas Gerais, Brazil; 2 Centre for Tropical, Environmental, and Sustainability Sciences, College of Marine and Environmental Sciences, James Cook University, Cairns, Queensland, Australia; 3 Department of Science, Information Technology, Innovation and the Arts,Queensland Herbarium, Brisbane, Queensland, Australia; 4 School of the Environment, University of Technology, Sydney, New South Wales, Australia; Chinese Academy of Forestry, CHINA

## Abstract

Understanding how tropical rainforest trees may respond to the precipitation extremes predicted in future climate change scenarios is paramount for their conservation and management. Tree species clearly differ in drought susceptibility, suggesting that variable water transport strategies exist. Using a multi-disciplinary approach, we examined the hydraulic variability in trees in a lowland tropical rainforest in north-eastern Australia. We studied eight tree species representing broad plant functional groups (one palm and seven eudicot mature-phase, and early-successional trees). We characterised the species’ hydraulic system through maximum rates of volumetric sap flow and velocities using the heat ratio method, and measured rates of tree growth and several stem, vessel, and leaf traits. Sap flow measures exhibited limited variability across species, although early-successional species and palms had high mean sap velocities relative to most mature-phase species. Stem, vessel, and leaf traits were poor predictors of sap flow measures. However, these traits exhibited different associations in multivariate analysis, revealing gradients in some traits across species and alternative hydraulic strategies in others. Trait differences across and within tree functional groups reflect variation in water transport and drought resistance strategies. These varying strategies will help in our understanding of changing species distributions under predicted drought scenarios.

## Introduction

One of the major threats to earth’s biodiversity are precipitation extremes predicted in future climate change scenarios [1]. In particular, tropical forests are likely to experience more frequent and severe droughts and increased aridity [2, 3], which can result in high levels of hydraulic stress in trees, loss of biomass and increased mortality [4, 5]. Drought is also likely to shape species’ distributions in tropical forests [6, 7]. The resilience of tropical forests to such events will therefore depend collectively on the strategies trees use to transport water and respond to water deficits.

Tree water transport can be studied by measuring rates of volumetric sap flow and sap velocities [8, 9]. Over the past decade, researchers have been adopting an increasingly multidisciplinary approach to study plant water transport incorporating the measurement of several stem and leaf traits [10, 11]. Such functional traits encompass morphological and physiological features that regulate the ecological functioning of a species [12]. Leaf area and leaf length to breadth ratio (i.e. leaf slenderness) for instance, determines the available area for transpiration [13]. Other leaf traits such as leaf mass per area are strongly correlated with key biological processes, including growth rate [14] and photosynthetic capacity [15], which in turn may be directly or indirectly related to plant water use [16].

Wood density is a key functional trait that is positively correlated with drought resistance [17] by increasing the strength of fibres associated with vessels [18], and by increasing the resistance of sapwood to xylem embolism [19]. However, the universality of this relationship is in question as even within a single biome, for example, rainforests, trees exhibit a wide range of wood densities [20, 21]. Rather, differences in wood density may relate more to adjustments in vessel characteristics such as vessel cross-sectional size, density and fractions (i.e. frequency and cross-sectional area occupied by open vessel spaces respectively per unit area) [21, 22].

More important than having a modulating effect on wood density, stem vessel size distribution, density and fractions affect the hydraulic conductivity of xylem [23, 24]. The different combinations of vessel sizes and densities can also reflect different strategies used to deal with water deficit [25]. Other anatomical traits such as the amount and arrangement of parenchyma in wood are also increasingly being recognized for their functional significance relating to drought adaptation [21, 25, 26]. Sapwood area and the ratio of sapwood to leaf areas (i.e. Huber values) also have important roles in modulating water transport and tree growth [27, 28]. Collectively, these stem traits are important structural aspects of the hydraulic architecture of a plant [29].

Since both leaf and stem traits are involved in plant water relations, understanding how trees may respond to water deficit requires an understanding how these traits vary across species [30, 31]. Given the high tree species richness in the tropics, we hypothesise a commensurate variety of trait strategies involved in the common function of water transport and drought resistance. In recent years, ecologists have used multivariate methods to examine various aspects of these relationships [22, 32] and to characterise water use trait strategies across species [11, 26, 31].

An ongoing rainfall exclusion experiment in a permanent plot of lowland tropical rainforest in northern Australia provided an opportunity to study the water transport trait strategies of tropical trees. The study species are being monitored *in situ* with sap flow sensors to determine their rates of water use prior to the implementation of rainfall exclusion. Understanding how water transport trait strategies vary between species and how this affects sap flow will provide an important context for the rainfall-exclusion study. We therefore characterized and examined the trait strategies related to water transport in eight co-occurring species of lowland tropical rain forest trees.

## Materials and Methods

### Ethics Statement

Permission to sample vegetation was not necessary as the study site was on land owned by the James Cook University. Internal approval from James Cook University was granted for performing the experiment on the property. No protected species were sampled.

### Study site and species

Our study site is located in a one-hectare permanent plot in lowland tropical rainforest at the Daintree Rainforest Observatory, Cape Tribulation (16°06′20″S, 145°26′40″E) in north-eastern Australia. We selected seven common eudicot and one monocot palm (>8 tree stems/ha >10 cm DBH), representing different functional groups [33] (Table 1). These species belong to different genera and families, thereby preventing bias due to close phylogenetic relatedness [34]. For brevity, we henceforth refer to our study species by their genus. We sampled four mature individuals (>60% potential height) of each species, with the exception of *Castanospermum* for which resources limited sampling to two individuals. Our sample selection was complemented with a canopy crane assessment to ensure that we selected individuals with well-illuminated crowns, that were not overtopped by other species, and which had limited liana load. In the case of *Myristica*, which is an understorey tree, all the individuals we selected were partially overtopped by canopy trees (60% to 70% crown exposed). However the variation in crown exposure of the four selected individuals was minimal, and in all cases well-illuminated branches were accessible by the crane.

**Table 1:** Tree species used in the study, their functional groups and high-order trait characteristics.

Species	Family	Functional group	DBH (cm) (mean, ±SD)	Height (m) (mean, ±SD)	Wood density (g cm^-3^) (mean, ±SD)
*Alstonia scholaris* (L.) R.Br.	Apocynaceae	Early successional, canopy tree	23.73, ±3.7	19.3, ±3.7	0.33, ±0.03
*Elaeocarpus angustifolius* Blume	Elaeocarpaceae	Early successional, canopy tree	32.13, ±11.84	20.9, ±5.1	0.49, ±0.07
*Argyrodendron peralatum* (F.M.Bailey) Edlin ex J.H.Boas	Malvaceae	Mature-phase, canopy tree	35.92, ±3.52	28.9, ±4.5	0.81, ±0.07
*Castanospermum australe* A.Cunn. ex Mudie	Fabaceae	Late successional to mature-phase, canopy tree	85.25, ±59.11	31.4, ±2.3	0.62, ±0.01
*Endiandra microneura* C.T.White	Lauraceae	Mature-phase, canopy tree	32.93, ±13.74	22.4, ±4	0.68, ±0.05
*Myristica globosa* (Warb.) W.J.de Wilde	Myristicaceae	Mature-phase, subcanopy tree	33.5, ±4.55	21.7, ±4.3	0.5, ±0,02
*Syzygium graveolens* (F.M.Bailey) Craven & Biffin	Myrtaceae	Mature-phase, canopy tree	54.22, ±8.3	27.9, ±2.2	0.56, ±0.06
*Normanbya normanbyi* (W.Hill) L.H.Bailey	Arecaceae	Mature-phase, subcanopy palm tree	14.68, ±0.59	16.6, ±1.4	1.46, ±0.02

Functional grouping and canopy occupancy of the species is based on Goosem and Tucker (p.35 in [33]) and field observation.

### Traits

We measured physiological, morphological and anatomical traits related to plant water use and growth (Table 2).

**Table 2:** Traits evaluated in the current study and their hypothesized ecological relevance to tree water use.

Traits	Unit	Ecological relevance
Maximum sap flow rate	cm^3^ hour^-1^	Sap conductance
Maximum sap flow velocity	cm hour^-1^	Sap conductance
Annual basal area increment	cm^2^ year^-1^	Plant growth
Sapwood area	Cm^2^	Sap conductance
Sapwood vessel area	μm^2^	Sap conductance
Sapwood vessel density	mm^2^	Sap conductance
Sapwood vessel fraction	No unit	Sap conductance
Sapwood vessel diameter	μm	Sap conductance
Theoretical specific conductivity	kg s^-1^ MPa^-1^	Sap conductance
Vulnerability index	No unit	Sap conductance and susceptibility to vessel cavitation
Leaf area	cm^2^	Transpiration and photosynthesis
Leaf dry matter content	mg mg^-1^	Structural support and leaf water storage
Leaf mass per unit area	mg mm^-2^	Structural support and growth
Leaf slenderness	cm cm^-1^	Control of water status
Leaf thickness	mm	Resistance to leaf drying
Huber value	cm^2^ cm^-2^	Sap conductance and transpiration
Min Leaf water potential	MPa	Physiological measure of the leaf water status
Max Leaf water potential	MPa	Physiological measure of the leaf water status
Carbon isotope ratio	‰	Physiological measure of intrinsic water-use-efficiency in leaves
Intrinsic water-use-efficiency	μmol mol^-1^	Physiological measure of intrinsic water-use-efficiency in leaves

We obtained sap flow measurements by the heat ratio method [35] using commercially-available sap flow meters (Model: SFM1; ICT International) [36]. The sap flow meters were installed at 1.3 m height on the bole, with the exception of buttressed trees (*Argyrodendron*) on which the devices were installed 50 cm above the buttress. Sap flow data was used for a four to nine day time window at the end of the dry season in November 2013 as this coincided with a period where all equipment was functioning simultaneously. The total rainfall for the nine-day period was 27 mm, and there was no rainfall recorded for a week prior to this. Meteorological data was obtained from the Bureau of Meteorology [37] for the Cape Tribulation Store weather station (no. 31012), which is < 1 km northeast of the study site. For each individual, we computed the mean maximum rates of sap flow and sap velocities over four to nine complete days.

As a measure of tree growth, we calculated the mean annual basal area increment (BA_*i*_) of our study individuals using a 12-year (2001–2015) dataset from the permanent one-hectare plot. BA_*i*_ was obtained by subtracting the most recent from the earliest basal area measurement available, and then dividing the difference with the number of years that had elapsed between those two measurements. We restricted BA_*i*_ comparisons to the seven eudicot species.

From the individuals sampled for sap flow, we measured stem and leaf functional traits, which are variously correlated with plant growth rate, light-use-efficiency, water-use-efficiency and relative drought resistance [12] (Table 2).

Two cores, each *ca*. 5 cm long, were obtained with an increment corer from each individual tree at breast height. We measured using callipers the depth of sapwood, which was determined visually by a colour change in the sapwood/heartwood interface. We used this to calculate sapwood area by subtracting the cross-sectional area of the non-sapwood area from that of the trunk (excluding bark). “Sapwood area” for *Normanbya* was calculated from the entire cross-sectional area of the palm stem at 1.3 m DBH, excluding the bark. Due to the hard wood, coring for sapwood samples was also not feasible for *Normanbya*. We therefore collected wood wedge samples at 1.3 m DBH from *Normanbya* individuals of a similar diameter to those used in sap flow measurements. The sapwood samples were sanded and polished with increasing grits of sanding paper until anatomical structures were visible. Sapwood vessels from the first two centimeters of sapwood towards the bark were examined with a stereo microscope (30x magnification; Nikon SMZ 745T) and photographed (Nikon DS-Fi2). On the digital photographs, vessels were measured for area (vessel area: VA) and density (vessel density: VD) using imaging software GIMP 2.8.10 [38].

From the vessel areas and cross sectional areas of digital images, we computed the vessel fraction (VF = VA × VD: total cross-sectional area used for sap transport). The idealized vessel diameters (V_*dia*_) was calculated for each species and we used the Hagen–Poiseuille equation to calculate the theoretical specific xylem hydraulic conductivity (henceforth theoretical specific conductivity) as: K_s_ = π/128ƞ*A*_cross sectional area_ x ΣD^4^, where ƞ is the viscosity (1.002 × 10^−9^ MPa s^-1^) and ρ is the density of water (998.23 kg m^-3^) respectively at 20°C [23]. We also derived a vulnerability index (VI = V_*dia*_/VD: susceptibility to cavitation) [39] for each individual. Wood density was measured on adjacent 5 to 10 mm length of sapwood core segments from the same cores sampled for the vessel measurements using the water displacement method [20]. For the palm *Normanbya*, we used wood wedge samples (see above).

We used the canopy crane to access leaf and branch material in the canopy. Leaf water potential (Ψ_L_) was measured at regular intervals from predawn (0500–0600 h) to 1950 h within a week in May 2014, using a pressure chamber [12]. The total rainfall for the week prior to collection dates was 52 mm, and there was no rainfall for a week prior to this period [37]. For our purposes, we only use the averages of the minimum and maximum Ψ_L_ as indicators of the leaf water status at the driest time of the day and resting state water relations respectively [12, 40]. Milky exudate in *Alstonia scholaris* obscured pressure chamber readings so we used literature Ψ_L_ values from a related species, *Alstonia macrophylla* Wall. [41]. Like *A*. *scholaris*, *A*. *macrophylla* is a fast growing rainforest successional species [42]. For leaf area, leaf slenderness (defined as the ratio of the leaf length to leaf breadth), leaf mass per area (LMA) and leaf dry matter content (LDMC), five to 20 replicates per individual of sun-exposed leaves were obtained from the tree canopy. For the compound-leaved *Castanospermum*, leaflets were taken to be the functional unit equivalent to leaves. Leaf areas were obtained from scans processed in imaging software GIMP. Leaf thickness was measured using a calliper, avoiding major veins. These leaves were weighed fresh and then weighed after drying 60°C for a week. LMA was the ratio of leaf dry weight to oven dried mass and LDMC was leaf dry mass divided by fresh mass. For leaf carbon isotope ratio (δ^13^C) determination, leaves were ground finely using a bead mill grinder and analysed at the Terrestrial Ecohydrology laboratory, School of the Environment, University of Technology, Sydney. We calculated the intrinsic water-use-efficiencies (WUE_i_) of each species from δ^13^C values after Werner et al. [43]. For Huber values we collected terminal branches with leaves and measured the total leaf areas of all the leaves on the branch, and also the cross sectional area of the cut branch. Huber values were then computed by dividing the branch sapwood area (excluding pith area) by leaf areas.

### Data Analyses

We analysed the data using both univariate and multivariate statistics. Variables were checked for normality and transformed where necessary before analysis. Univariate one-way ANOVAs were performed for each trait and significant differences between species were determined by Tukey HSD tests (confidence level of 0.05). From 30 trees, we identified major gradients in functional traits using Non-metric multidimensional scaling (NMDS) ordination [44]. To improve ordination performance, traits were standardized. Monte Carlo randomization tests (100 runs) were used to determine whether ordination axes explained significantly more variation than expected by chance. When testing for correlations between individual functional traits and ordination axes, Bonferroni-corrected alpha values were used to reduce the experiment-wise error rate (*p* = 0.1/28 = 0.00357).

We used linear mixed effects models (with Restricted Maximum Likelihood) to examine relationships between sap flow measures and annual basal area increment (response variables) as a function of leaf and stem traits. We used the two NMDS axes as fixed factors in the models to resolve the high levels of inter-correlations between the leaf and stem traits. Other fixed factors included in the saturated models were individual tree characteristics: height and DBH. Species were included as a random factor, accounting for seven tree species due to the exclusion of the palm *Normanbya*. All modelling was performed in R [45], using the package nlme [46] following a standard protocol for data exploration [47].

For each response variable, the most supported set of predictors was inferred using an Akaike’s information-theoretic approach [48] corrected for sample size, and the top five models which included >95% of Akaike weight are presented. To obtain a measure of relative support of each model combination, we also computed their Akaike weight *w*, which refers to the probability of a model combination being the best supported one among a given model set. Inferences were drawn based on all plausible models.

## Results

### Sapwood anatomical observations

**Fig 1:**
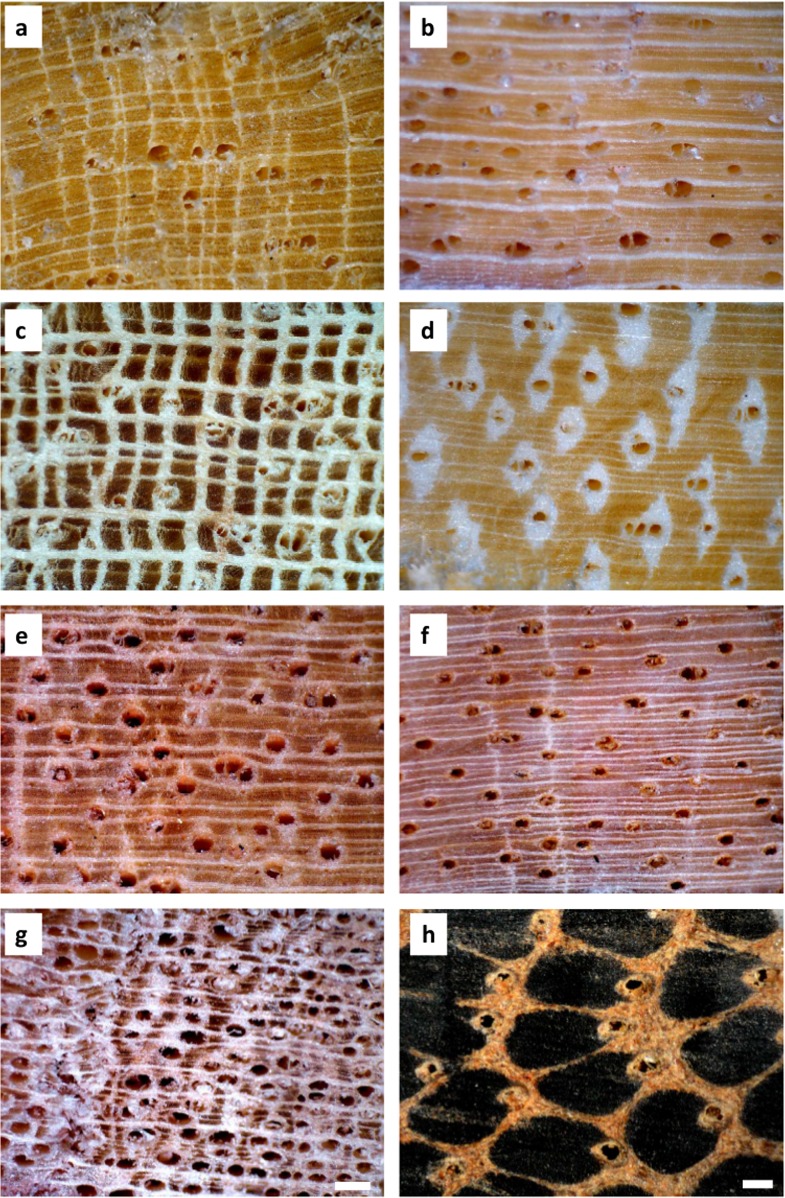
Contrasting wood anatomical features (a-h) of tropical lowland rainforest trees from Daintree, Australia. Early successional trees (a) *Alstonia scholaris* and (b) *Elaeocarpus angustifolius* with light coloured wood and scattered vessels; mature-phase trees (c) *Argyrodendron peralatum*, (d) *Castanospermum australe*, (e) *Endiandra microneura*, (f) *Myristica globosa*, and (g) *Syzygium graveolens* with varying vessel and parenchyma arrangements, and; palm (h) *Normanbya normanbyi* with dark fiber bundles. The white scale bar in *Syzygium* equal 0.2 mm and serves for all images except for *Normanbya* where it equals 0.25mm.

With the exception of the palm, all the study species are eudicot trees with diffuse porous wood, and had light-coloured parenchyma tissue appearing either as bands of varying widths or in patches. Early-successional trees (*Alstonia*, *Elaeocarpus*; Fig 1A and 1B) have scattered vessels with relatively thin radial parenchyma bands. Mature-phase trees differ drastically in wood anatomy, particularly with reference to patterns of parenchyma-vessel association and vessel density. *Endiandra* (Fig 1E) and *Myristica* (Fig 1F) have solitary vessels, and irregularly-spaced bands of radial parenchyma. *Argyrodendron*, *Castanospermum* and *Syzygium* (Fig 1C, 1D and 1G respectively) has vessels distinctively associated with parenchyma. In the case of *Castanospermum*, the vessels are consistently encapsulated in patches of abundant parenchyma tissue (aliform parenchyma) which are sometimes confluent (Fig 1C). Notably also, *Syzygium* has the most densely-packed vessels among all the study species (Fig 1G). The vascular system of the single palm *Normanbya* differs drastically from those of the other functional groups, consisting of dark scattered vascular bundles within a matrix of light-coloured ground tissue. Each vascular bundle is comprised largely of dense fibres with a single large vessel is situated at the distal end (into trunk) (Fig 1H).

### Sapflow and Relative Growth

**Fig 2:**
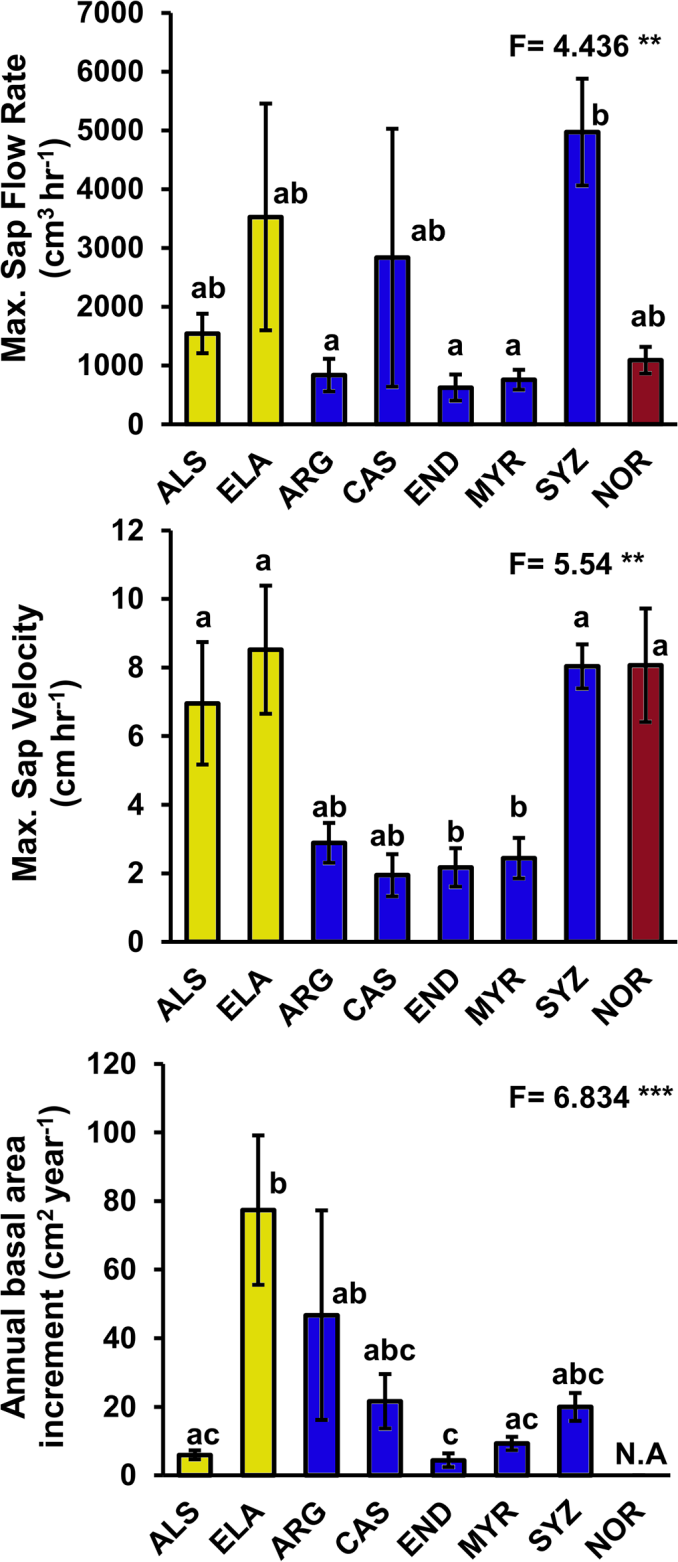
Means (±S.E) of sap flow measures and annual basal area increment of tropical lowland rainforest tree species from Daintree, Australia. Species codes are as follows for early successional (yellow bars), mature-phase (blue bars) and palm species (maroon bars): *Alstonia scholaris* (ALS); *Elaeocarpus angustifolius* (ELA); *Argyrodendron peralatum* (ARG); *Castanospermum australe* (CAS); *Endiandra microneura* (END); *Myristica globosa* (MYR); *Syzygium graveolens* (SYZ); *Normanbya normanbyi* (NOR). The palm species NOR was excluded from the annual basal area increment graph as it does not exhibit secondary growth comparable with the other seven eudicot trees. One-way ANOVA F-values are given and significance levels are indicated by asterisks as follows: *P*<0.05*, <0.01**, <0.001***. Numerator degrees of freedom and denominator error degrees of freedom are 7 and 22 respectively for sap flow measures and 6 and 19 respectively for annual basal area increment. Significant differences between species are indicated by different letters (Tukeys HSD, *p* < 0.05).

We observed differences among functional groups in maximum sap velocity but not within sap flow rate or annual basal area increment (Fig 2; S1 Table). Maximum sap velocity showed two distinct responses with low averages for the mature-phase species (*Argyrodendron*, *Castanopermum*, *Endiandra* and *Myristica*) and high sap velocities for the early-successional (*Alstonia*, *Eleaocarpus*), the palm and mature-phase *Syzygium* (One-way ANOVA: *F*_7,22_ = 5.54, *p* < 0.001). High and low averages for max sap flow rates were recorded for both early successional and mature-phase species (*F*_7,22_ = 4.436, *p* = 0.0033) respectively. Annual basal area increments were similar across all functional groups, except for remarkably high values for one early-successional tree (*F*_6,19_ = 6.834, *p* < 0.001).

### Stem traits

**Fig 3:**
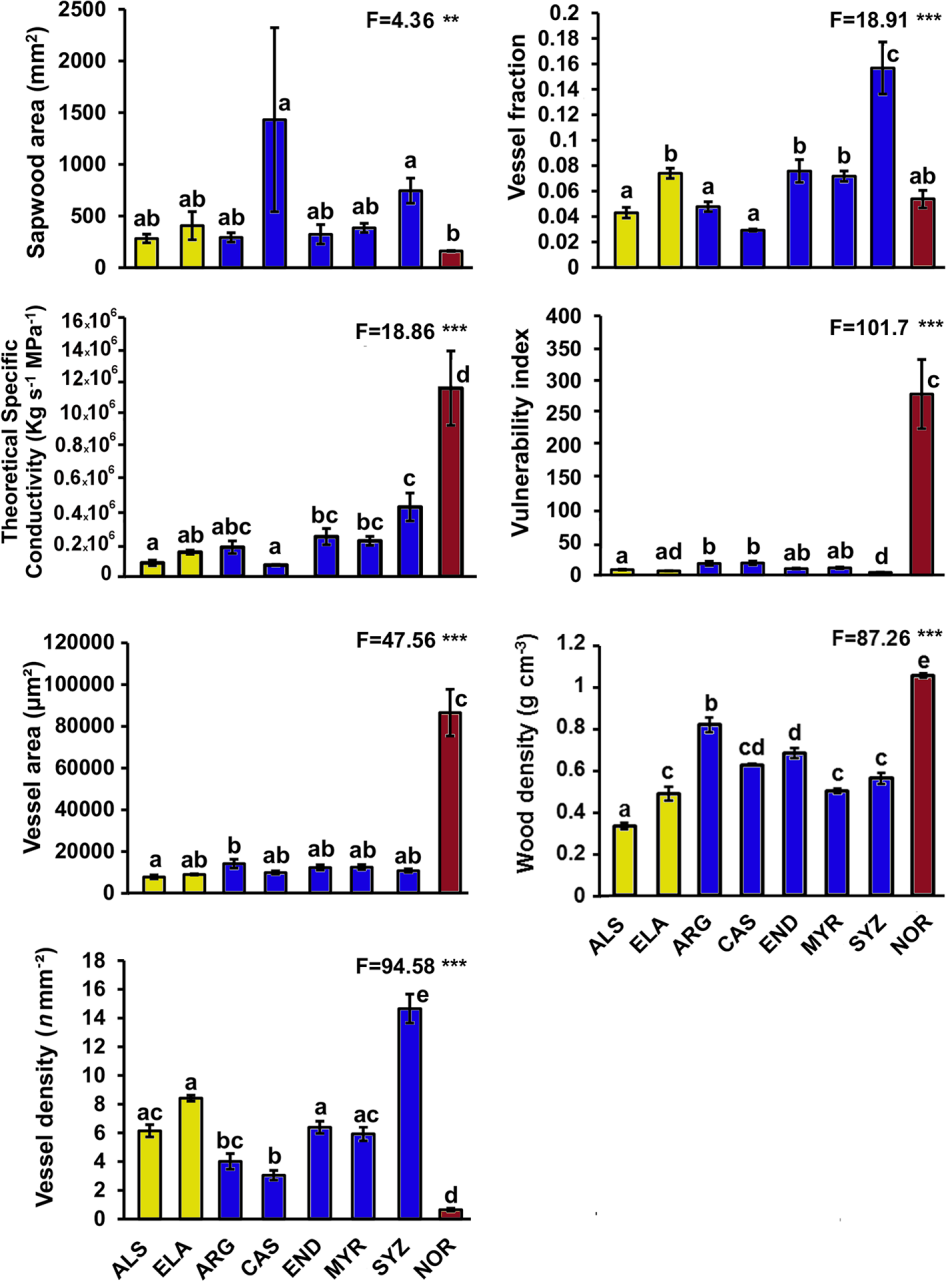
Means (±S.E) of stem traits of tropical lowland rainforest tree species from Daintree, Australia. Species and colour codes follows Fig 2. One-way ANOVA F-values are given and significance levels are indicated by asterisks as follows: *P*<0.05*, <0.01**, <0.001***. Numerator degrees of freedom and denominator error degrees of freedom are 7 and 22 respectively for all variables. Significant differences between species are indicated by different letters (Tukeys HSD, *p* < 0.05).

We observed differences among functional groups with the single palm species responding significantly differently from the early-successional and mature-phase trees in the following traits (Fig 3; S1 Table): vessel area (*F*_7,22_ = 47.56, *p* < 0.001), vessel density (*F*_7,22_ = 94.58, *p* < 0.001), vulnerability index (*F*_7,22_ = 101.7, *p* < 0.001), and theoretical specific conductivity (*F*_7,22_ = 18.86, *p* < 0.001). Not surprisingly, wood density differed significantly among early-successional, mature-phase and the palm species (*F*_7,22_ = 87.26, *p* < 0.001). Sapwood area reflected tree size but no functional groups pattern (*F*_7,22_ = 4.36, *p* = 0.004) and Huber value showed no difference across species (*F*_7,22_ = 1.256, *p* = 0.316) and is not included in Fig 3.

### Leaf traits

**Fig 4:**
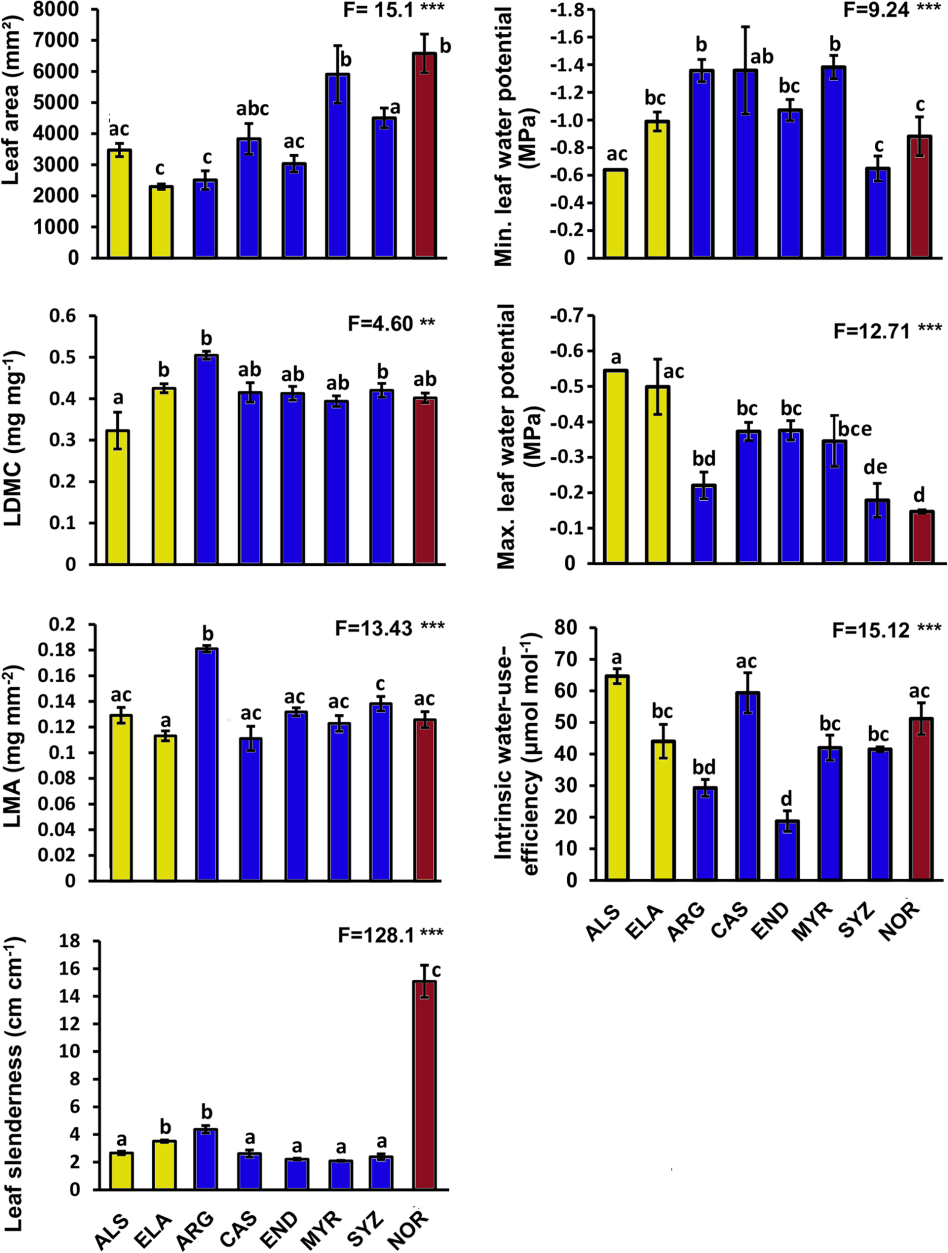
Means (±S.E) of leaf traits of tropical lowland rainforest tree species from Daintree, Australia. Species and colour codes follows Fig 2. One-way ANOVA F-values are given and significance levels are indicated by asterisks as follows: *P*<0.05*, <0.01**, <0.001***. Numerator degrees of freedom and denominator error degrees of freedom are 7 and 22 respectively for all variables. Significant differences between species are indicated by different letters (Tukeys HSD, *p* < 0.05).

We observed few differences among functional groups in leaf traits (Fig 4; S1 Table). One mature-phase species had a significantly higher LMA than all other species (*F*_7,22_ = 13.43, *p* < 0.001) and the single palm species distinctively different from other functional groups with respect to leaf slenderness (*F*_7,22_ = 128.1, *p* < 0.001). Leaf thickness showed no difference across species (*F*_7,22_ = 0.553, *p* = 0.785) and is not included in Fig 4.

### Coordination among functional traits and sap flow response modelling

**Fig 5:**
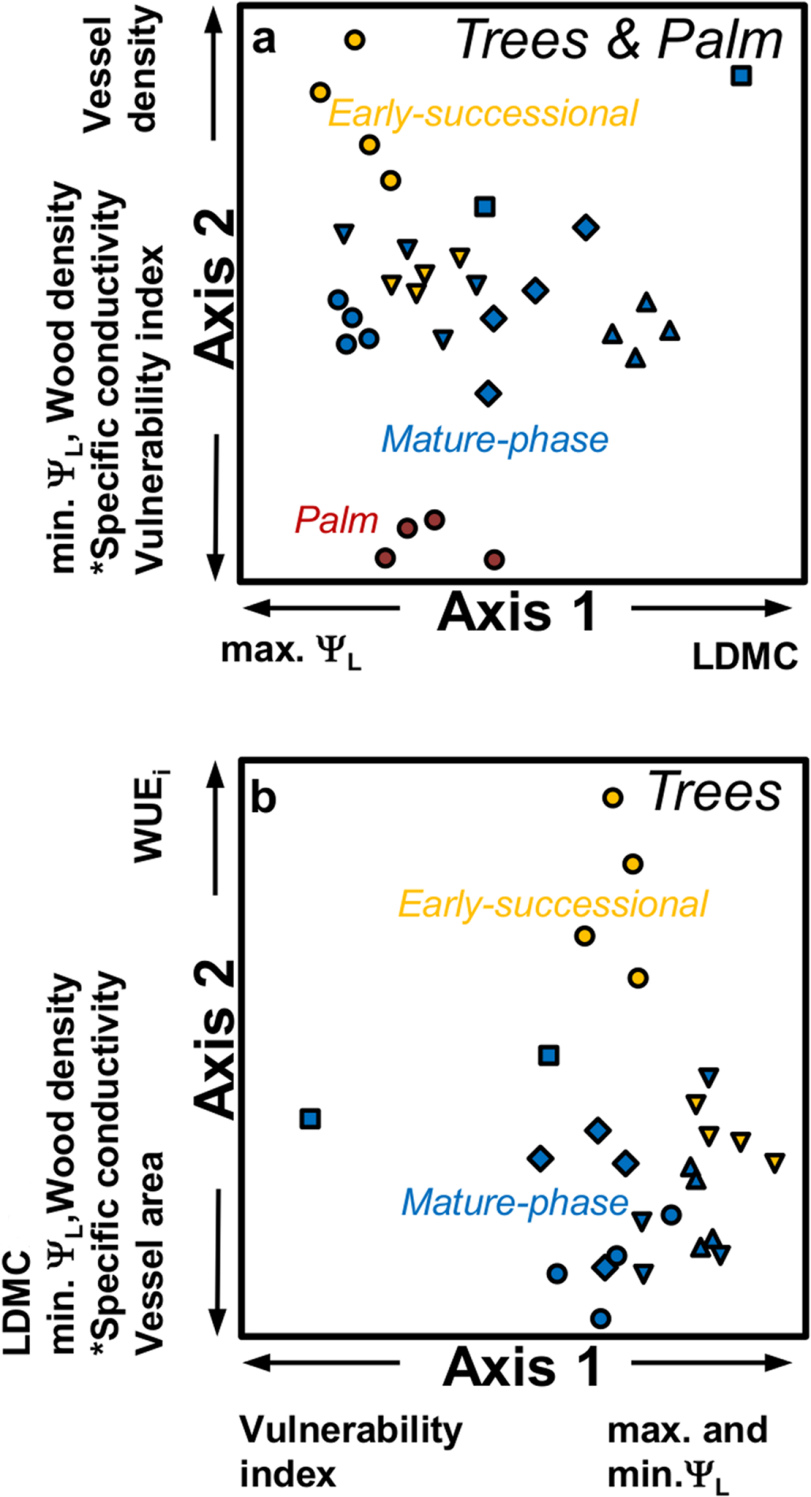
Non-metric multidimensional scaling ordinations of traits of tropical lowland rainforest species including (a) and excluding (b) the palm species. Traits and their abbreviations (in parentheses) include: vessel area, vessel density, *theoretical specific conductivity, wood density, leaf dry matter content (LDMC), leaf slenderness, minimum and maximum leaf water potentials (min Ψ_L_, max Ψ_L_ respectively), intrinsic water-use-efficiency (WUE_i_) and vulnerability index. Species symbols are as follows: Secondary-successional species – *Alstonia scholaris* (yellow circles); *Elaeocarpus angustifolius* (yellow inverted triangles); Mature-phase species – *Argyrodendron peralatum* (blue circles); *Castanospermum australe* (blue squares); *Endiandra microneura* (blue inverted triangles); *Myristica globosa* (blue diamonds); *Syzygium graveolens* (blue triangles), and; Palm – *Normanbya normanbyi* (maroon circles). The arrows by the axes indicate significant positive or negative Pearson correlations between individual traits and axes.

We explored the coordination of functional traits across 30 rainforest trees (seven eudicot tree species and one palm species) using non-metric multidimensional scaling ordination analysis (NMDS). Two ordination axes collectively explained 86.5% of the total variation in the functional trait data set (Fig 5A). Axis 2, which captured 73.5% of the variation, clearly distinguished a gradient of early-successional, mature-phase and palm trees. This gradient showed the functional coordination of seven of the 14 water-use traits examined in this study (Fig 5A; Table 3), with low values for an early-successional species (*Alstonia*), moderate values for a group of mature-phase species (and one early-successional species) and high values for a palm species. The lack of interspecific variation among the five mature-phase species and one early-successional species along this axis is notable. Axis 1 explained 13% of the total variation and described a gradient among species that does not reflect obvious functional grouping but demonstrates an orthogonality between leaf volume (measured as LDMC) and maximum leaf water potential. Along the Axis 1 gradient, we observe interspecific clustering of trees within four mature-phase species, with the remaining four overlapping.

**Table 3:** Pearson correlations for functional traits with two ordination axes produced by Non-metric multidimensional scaling (NMDS).

Traits	NMDS with *Normanbya*		NMDS without *Normanbya*	
	Axis 1	Axis 2	Axis 1	Axis 2
Leaf dry matter content	**0.548**	-0.39	-0.004	**-0.773**
Leaf slenderness	0.061	**-0.753**	n.s	n.s
Leaf water potential (Max.)	**-0.669**	-0.105	**0.715**	0.41
Leaf water potential (Min.)	-0.297	**-0.715**	**0.646**	**-0.564**
Vessel area	0.011	**-0.862**	-0.14	**-0.779**
Vessel density	-0.23	**0.587**	**0.694**	-0.057
Vessel fraction	n.s	n.s	**0.608**	-0.478
Theoretical specific conductivity	-0.224	**-0.89**	0.37	**-0.721**
Wood density	0.485	**-0.798**	0.088	**-0.772**
Water-use-efficiency	n.s	n.s	-0.277	**0.691**
Vulnerability index	0.171	**-0.687**	**-0.665**	-0.153

Only traits exhibiting significant associations with at least one axis are listed. Values in bold were significant using a Bonferroni-corrected alpha value (*p* = 0.00357) (See also Fig 5A and 5B).

Palms lack sapwood and growth rings and are likely to be distinctly different from eudicot trees with respect to hydraulic architecture. Hence, we further explored the robustness of our functional coordination among traits, by considering only eudicot trees (26 individuals) in a second ordination analysis (Fig 5B). The second ordination explained 83% of the total variation in the data and showed remarkable similarities with the first (Fig 5B). Axis 2, distinguished a similar gradient from early successional to mature-phase species, capturing 53.5% of the total variation. We observed four of the seven functional traits that defined this gradient in the earlier ordination also coordinating and influencing the position of trees in this analysis (Table 3). Along this axis only an early successional species (*Alstonia*) showed interspecific variation, but most overlapped, demonstrating the natural variation within species and the importance of within species replication. Similarly, there was no interspecific variation detected among the species in Axis 1 which explained 29.5% of the variation and described a gradient of leaf water potential (maximum and minimum) and vessel fraction and density at one end, and vulnerability index at the other end. Interestingly, the individual tree identified as vulnerable is the largest individual on the one-hectare plot measuring 127 cm DBH.

Sap flow velocity and annual basal area increment were poorly predicted by stem and leaf traits as ordinated variables from NMDS and individual tree characteristics (DBH and height) when nested within species (Table 4). Sap flow velocity values were low and similar across four species and high and variable across three species (Fig 2), but these patterns were not reflected in the ordination gradients. With respect to annual basal area increment these were overall low and similar across six species and high in a single species (*Elaeocarpus*), also a pattern not reflected in the ordination axes or other functional traits examined.

**Table 4:** Top candidate models that predict mean maximum sap velocity and mean annual basal area increment across seven tropical lowland rainforest tree species in Daintree, northeast Australia.

Response variable	Intercept	NMDS axis 1	NMDS axis 2	DBH	AICc	ΔAICc	*w*	*w*_*1*_ */w*_*i*_
Sap velocity	0.690				-0.8	0.00	0.450	
	0.552		0.520		0.3	1.06	0.265	1.698
	0.657	0.073			1.9	2.67	0.118	2.246
	0.759			-0.044	3.1	3.86	0.065	1.815
	0.530	0.052	0.518		3.3	4.06	0.059	1.102
*(Σw – top 5 models)*	*0*.*957*	*0*.*177*	*0*.*324*	*0*.*065*				
Basal area increment	1.187				32.7	0.00	0.315	
	1.365		-0.673		33.5	0.75	0.217	1.452
	0.341			0.536	33.9	1.17	0.176	1.233
	1.143	0.098			34.1	1.35	0.160	1.100
	-0.437	0.842		0.786	34.5	1.74	0.132	1.212
*(Σw – top 5 models)*	*1*.*000*	*0*.*292*	*0*.*217*	*0*.*308*				

AICc refers to Akaike Information criterion corrected for small sample size, **∆**AICc to the difference between each model’s AICc and the minimum AICc found, *w* to Akaike weights, and *w*_*1*_/*w*_*i*_ to evidence ratios where *w*_*1*_ is the Akaike weight of the best fitting model. Akaike weights may be interpreted as relative model probabilities. Models with a higher evidence ratio are less likely to be the best model. The summed weights (Σw) for predictors is the relative likelihood that the predictor should form part of the model [48]. The top candidate models include ≥95% of Akaike weights.

## Discussion

In the wet lowland rainforests of northern Australia, we found a coordination of stem and leaf water-use characteristics that followed an ecological gradient in our species. We found seven traits (minimum leaf water potential, leaf slenderness, wood density, theoretical specific conductivity, vessel area and density, and vulnerability index) that were positively correlated with the gradient from early successional to mature-phase species to palm trees. Importantly among the five mature-phase species we studied, there was considerable trait variation within species and hence a marked lack of interspecific variation among species, which suggests the importance of replication within species for eco-physiological studies. Despite detectable differences in relative growth rates and sap flow values among some species, our data on the stem and leaf functional traits important for water transport were poor predictors in explaining this variation.

### Various stem and leaf trait associations effect water transport in trees

Our species and individual trees were selected to span a range of wood density and tree size values in order to detect trait variations. We found large overlaps across species in maximum rates of sap flow and velocities across species, which may be expected for plants growing in the same environment [49, 50, 51]. Our linear mixed effects models showed that tree features and the functional coordination of stem and leaf traits were not good predictors of sap flow velocity and basal area incremental growth. One explanation for this lack of predictability is that the variation exhibited within species was greater or equal to the variation between species.

Our NMDS however, revealed various gradients and associations of traits that may reflect contrasting strategies in water transport traits. For instance, leaf carbon isotope ratios reflect a plant’s intrinsic water-use-efficiency (WUE_i_) [43], and can differentiate early-successional from mature-phase species [52]. Indeed, WUE_i_ appears to segregate early-successional species *Alstonia* from the clustering of mature-phase species (*Argyrodendron*, *Endiandra* and *Syzygium*) (Fig 5).

In their multivariate analyses, Worbes et al. [11] showed leaf δ^13^C as having an analogous influence as vessel area, whereas we found an opposite relationship between vessel area and WUE_i_ (based on leaf δ^13^C). This disparity could reflect intrinsic differences in the adaptations to water deficit between the drier tropical forest type examined by Worbes et al. [11] and the humid tropical forest in the current study. In addition, Worbes et al. [11] sampled deciduous species, which were not represented in our sampled species.

A further potential coordination of leaf and stem traits was the relationships among leaf volume (dry matter content), minimum leaf water potential and theoretical specific conductivity from our ordinations. Similar reports of coordination between the stem and leaf economic spectra, was reported in Méndez-Alonzo et al. [32] and Kröber et al. [31] in tropical dry forest and in a common garden experiment respectively. We hypothesised that a high vessel area or vessel density may be associated with large stem water storage capacity, which may in turn lead to a less negative maximum and minimum water potential (Ψ_L_). For tropical deciduous species, Worbes et al [11] reported that vessel size and density are linked to the most negative Ψ_L_ values as the driving force for water transport. Other traits such as leaf vein [53] and stomatal densities [54] may contain more relevant information for establishing the link between the leaf and stem economic spectra and therefore deserve measuring in future studies.

### Trait strategies for water transport and drought resistance

Water transport and drought resistance involves a complex interplay of physiological processes [40, 55, 56], intrinsic tree architectural factors (e.g. xylem tapering) [57] and environmental factors (e.g. soil moisture, seasonality, vapour pressure deficits) [58] that we were unable to fully account for or measure. Nevertheless, we can summarize from the available data the water transport strategies and discuss empirically the potential drought resistance of our study species with relevance to their functional groups.

The early-successional species (*Alstonia*, *Elaeocarpus*) have relatively high WUE_i_ and low VIs, which suggests a relatively high drought tolerance relative to the other study species. These trait combinations may also allow these species to be in exposed habitats experiencing larger vapour pressure deficits (VPDs) whilst minimizing embolism risk. These species also tended to have higher maximum sap velocities, possibly reflecting their exposure to larger VPDs and light levels in more exposed environments characteristic of early-successional species.

The studied mature-phase species exhibited some observable variations in trait strategies. The mature-phase tree *Castanospermum* had intermediate wood density, high WUE_i_ values, the largest sapwood area, and was associated with the highest VIs of the eudicot trees. Conspicuously, the wood anatomy of *Castanospermum* was unique in having significant portions of parenchyma surrounding the vessels and this appears to be a common trait in trees from the Fabaceae [59, 60]. Borchett and Pockman [61] suggest that such anatomical features buffer the stem from abrupt water pressure changes during water loss or gain. Therefore, the high VI in *Castanospermum* may be compensated by having large quantities of sapwood water storage that can be utilized to repair embolisms [56, 62, 63], or to mitigate against the development of low xylem water potentials. Such a feature suggests a drought-avoiding rather than drought-resisting strategy for this species [21].

The mature-phase canopy species *Endiandra* and *Argyrodendron* had among the lowest annual basal area increments, relatively low sap velocities and maximum rates of sap flow, low WUE_i_ values, and relatively high VIs. This reflects a potentially low drought resistance. However, the conspicuous vessel-parenchyma association in *Argyrodendron* suggests that this species has higher water storage and possibly better ability to cope with drought than *Endiandra*. The sub-canopy tree *Myristica* had relatively low sap velocities and annual basal area increment, probably reflecting the low-light environment and low vapour pressure deficit that occurs in the sub-canopy of closed forests. However, it has intermediate WUE_i_ values and VIs, which might reflect the reduced evaporative demands [64] of being in the forest sub-canopy.

Surprisingly, we found a relatively high sap velocity and maximum rate of sap flow, moderate WUE_i_, and also extremely low VI (due to the high vessel packing per unit area) and the largest vessel density and vessel fraction in the mature-phase tree *Syzygium*. Such low VIs would be expected to appear in taxa of xerophytic environments [24] but ironically in *Syzygium*, this high vessel packing per unit area may confer greater physiological safety to drought than one with wider and fewer vessels. Such a strategy could work by restricting air embolisms to smaller and more localized sections of the sap flow column in the event of in high VPDs during a drought event [24, 65]. However, this may also compromise the species hydraulic efficiency via increased resistance to water transport [17, 66].

Our only sample of a palm, *Normanbya*, had stem, vessel, and leaf traits that differed drastically from species of other functional groups. From these observations and on the basis of well documented differences between the hydraulic architecture of palm and eudicot trees [67], we can infer a drastically different water transport and drought resistance strategy for *Normanbya* from our other eudicot study species. Unlike eudicot trees, palms lack of sapwood and growth rings, and the arrangement of the vascular system is predetermined [68]. The relatively high WUE_i_, high sap velocity and large vessel areas relative to our eudicot study species, suggests that *Normanbya* has a very efficient hydraulic system to maintain a large conducting capacity with a minimal vascular investment [67], especially where water is not limiting. However, these characteristics also suggest that this species will be vulnerable to droughts.

## Conclusion

Increased incidence and durations of drought are predicted scenarios for the future of lowland tropical rainforest, and underpins the importance of understanding the strategies that rainforest trees may use to cope with such conditions. Studying trees representing different functional groups, we found, with some differences, little variation in maximum sap velocities and rates of sap flow across species. We have demonstrated however within and across functional groups, that tropical lowland rainforest trees exhibit variable trait strategies for water transport. This is achieved through various associative extents in stem and leaf traits. In particular, quantitative wood anatomical features (vessel areas and densities) have a bearing on plant water transport and should therefore be examined as an informative trait in field-based ecophysiological studies involving the measurement of sap flow. Also, quantitative studies on vessel traits in conjunction with physiological measures can provide useful metrics for estimating a species drought resistance. This information will serve as an important context for an ongoing rainfall exclusion experiment in place, and will complement our understanding of changing species distributions of rainforest trees under various climate change scenarios.

## Supporting Information

S1 Table Sap flow, growth, and leaf and stem trait values of eight tropical lowland rainforest tree species, Daintree, Australia. (DOCX)
