# Supplementary material for: Functional Traits and Water Transport Strategies in Lowland Tropical Rainforest Trees
Source: PLoS One. 2015 Jun 18;10(6):e0130799. doi: 10.1371/journal.pone.0130799 (PMC4472991; doi:10.1371/journal.pone.0130799)
Supplement: S1 Table — (DOCX) [file pone.0130799.s001.docx]

Supporting Information

**Table S1.** **Sap flow, growth, and leaf and stem trait values of eight tropical lowland rainforest tree species, Daintree, Australia.**

| **Species** | **Code** | **DBH** | **HT** | **SF** | **SV** | **BA_i_** | **HV** | **SA** | **TSC** | **VA** | **VD** | **VF** | **VI** | **WD** | **LA** | **LDMC** | **LMA** | **LS** | **LT** | **ψ_min_** | **ψ_max_** | **δ^13^C** | **WUE_i_** |
| --- | --- | --- | --- | --- | --- | --- | --- | --- | --- | --- | --- | --- | --- | --- | --- | --- | --- | --- | --- | --- | --- | --- | --- |
| ALS | 613 | 26.7 | 22.2 | 2120.7 | 6.57 | 4.47 | 58.8 | 386.10 | 1129907 | 9016.7 | 5.61 | 0.05 | 9.54 | 0.29 | 3557.3 | 0.229 | 0.120 | 2.76 | 0.32 | -0.6 | -0.5 | -29.1 | 61.3 |
| ALS | 205 | 20.6 | 16.2 | 996.8 | 5.54 | 4.42 | 32.6 | 216.39 | 1135069 | 9454.0 | 6.97 | 0.05 | 7.87 | 0.35 | 3354.0 | 0.399 | 0.122 | 2.94 | 0.46 | -0.6 | -0.5 | -28.8 | 64.6 |
| ALS | 137 | 27.1 | 22.7 | 927.7 | 3.71 | 5.14 | 60.6 | 311.14 | 552013.1 | 5736.8 | 6.77 | 0.04 | 6.31 | 0.36 | 3996.8 | 0.398 | 0.147 | 2.37 | 0.32 | -0.6 | -0.5 | -29.1 | 61.4 |
| ALS | 478 | 20.5 | 15.9 | 2134.9 | 12.01 | 9.88 | 11.4 | 214.97 | 526600.7 | 6379.5 | 5.23 | 0.03 | 8.62 | 0.33 | 2984.4 | 0.266 | 0.128 | 2.60 | 0.36 | -0.6 | -0.5 | -28.2 | 71.4 |
| ELA | 337 | 35.4 | 19.9 | 1903.7 | 5.36 | 104.29 | 43.4 | 417.13 | 1801981 | 9141.0 | 8.71 | 0.08 | 6.19 | 0.54 | 2119.6 | 0.393 | 0.110 | 3.62 | 0.29 | -0.9 | -0.1 | -30.7 | 44.6 |
| ELA | 316 | 46.9 | 28 | 9294.1 | 13.93 | 123.76 | 36.3 | 785.67 | 1619844 | 8814.9 | 8.42 | 0.07 | 6.29 | 0.49 | 2519.1 | 0.441 | 0.108 | 3.70 | 0.53 | -1.0 | -0.2 | -30.2 | 50.3 |
| ELA | 937 | 19.3 | 16 | 1172.5 | 7.22 | 32.69 | 11.4 | 176.55 | 1237662 | 7985.4 | 7.84 | 0.06 | 6.43 | 0.52 | 2206.6 | 0.431 | 0.125 | 3.38 | 0.31 | -0.9 | -0.3 | -30.0 | 52.5 |
| ELA | 928 | 26.9 | 19.7 | 1738.4 | 7.57 | 48.62 | 65.3 | 246.07 | 1349324 | 9380.1 | 8.71 | 0.08 | 6.27 | 0.39 | 2336.8 | 0.436 | 0.110 | 3.39 | 0.18 | -1.2 | -0.2 | -32.1 | 28.8 |
| ARG | 214 | 32.2 | 34.8 | 250.1 | 1.93 | 137.28 | 11.8 | 184.58 | 2100811 | 15189.5 | 4.35 | 0.04 | 15.97 | 0.85 | 2485.6 | 0.477 | 0.174 | 4.66 | 0.22 | -1.6 | -0.4 | -31.5 | 35.3 |
| ARG | 517 | 36.8 | 25.2 | 1516.9 | 4.51 | 4.91 | 28.6 | 372.33 | 922477 | 8291.3 | 5.42 | 0.05 | 9.48 | 0.79 | 1681.0 | 0.519 | 0.185 | 4.77 | 0.41 | -1.2 | -0.4 | -31.8 | 32.3 |
| ARG | 604 | 34.3 | 25.6 | 561.4 | 2.18 | 17.35 | 46.0 | 252.10 | 1477978 | 13883.5 | 3.10 | 0.04 | 21.47 | 0.88 | 3080.0 | 0.506 | 0.182 | 3.57 | 0.28 | -1.3 | -0.3 | -32.5 | 25.0 |
| ARG | 73 | 40.4 | 29.9 | 1025.7 | 2.93 | 27.27 | 10.8 | 362.26 | 2731606 | 18586.3 | 3.19 | 0.06 | 24.08 | 0.72 | 2781.3 | 0.516 | 0.184 | 4.49 | 0.54 | -1.4 | -0.4 | -32.5 | 24.5 |
| CAS | 189 | 43.5 | 29.7 | 641.6 | 1.33 | 29.54 | 40.9 | 541.40 | 663108.5 | 8892.0 | 3.39 | 0.03 | 15.71 | 0.63 | 4327.2 | 0.391 | 0.121 | 2.88 | 0.31 | -1.0 | -0.4 | -28.7 | 65.8 |
| CAS | 518 | 127.1 | 33 | 5026.5 | 2.56 | 13.70 | 35.4 | 2321.63 | 741326 | 10511.5 | 2.71 | 0.03 | 21.35 | 0.62 | 3336.0 | 0.439 | 0.102 | 2.36 | 0.30 | -1.6 | -0.6 | -29.9 | 53.0 |
| END | 634 | 14.7 | 16.4 | 190.2 | 2.17 | 1.14 | 18.9 | 70.67 | 1075774 | 8723.2 | 5.71 | 0.05 | 9.23 | 0.71 | 2683.3 | 0.375 | 0.128 | 2.09 | 0.34 | -0.9 | -0.4 | -32.8 | 21.2 |
| END | 957 | 46 | 23.4 | 290.3 | 0.71 | 2.89 | 26.7 | 414.57 | 2649465 | 13330.3 | 6.10 | 0.08 | 10.68 | 0.65 | 3550.7 | 0.452 | 0.139 | 2.39 | 0.34 | -1.1 | -0.4 | -33.7 | 12.1 |
| END | 220 | 40.5 | 24.7 | 1032.6 | 2.34 | 10.29 | 41.4 | 505.29 | 2675333 | 11888.2 | 7.64 | 0.09 | 8.05 | 0.63 | 2481.8 | 0.399 | 0.125 | 2.11 | 0.23 | -1.1 | -0.3 | -33.4 | 14.9 |
| END | 190 | 30.5 | 24.9 | 980.8 | 3.46 | 3.41 | 12.0 | 300.93 | 3467143 | 14695.7 | 6.10 | 0.08 | 11.22 | 0.72 | 3421.4 | 0.427 | 0.136 | 2.28 | 0.26 | -1.2 | -0.3 | -32.3 | 26.8 |
| MYR | 200 | 39.5 | 26.8 | 367.2 | 0.91 | 10.32 | 29.6 | 476.61 | 2221757 | 11270.1 | 7.06 | 0.08 | 8.48 | 0.52 | 4855.6 | 0.423 | 0.140 | 2.19 | 0.22 | -1.1 | -0.4 | -30.9 | 42.7 |
| MYR | 163 | 29.2 | 20.2 | 992.4 | 3.61 | 5.58 | 15.2 | 341.68 | 2317249 | 12695.5 | 5.81 | 0.07 | 10.95 | 0.47 | 8618.4 | 0.407 | 0.120 | 2.02 | 0.39 | -1.5 | -0.5 | -30.3 | 48.7 |
| MYR | 474 | 30.9 | 16.6 | 578.6 | 2.14 | 14.26 | 20.5 | 284.66 | 1470231 | 9868.8 | 6.10 | 0.06 | 9.19 | 0.50 | 5525.5 | 0.381 | 0.111 | 2.05 | 0.29 | -1.4 | -0.3 | -30.6 | 46.0 |
| MYR | 449 | 34.4 | 23.2 | 1090.6 | 3.09 | 6.99 | 21.3 | 438.43 | 2809135 | 15467.8 | 4.74 | 0.07 | 14.80 | 0.50 | 4631.2 | 0.365 | 0.121 | 2.08 | 0.22 | -1.5 | -0.2 | -32.0 | 30.6 |
| SYZ | 954 | 56.8 | 30.6 | 7136.6 | 9.25 | 28.36 | 16.5 | 1079.27 | 2897714 | 9499.8 | 13.0 | 0.12 | 4.24 | 0.52 | 4107.0 | 0.378 | 0.133 | 2.23 | 0.34 | -0.6 | -0.2 | -31.0 | 41.7 |
| SYZ | 36 | 48.9 | 28.6 | 2931.3 | 6.31 | 10.08 | 46.9 | 547.57 | 4423302 | 10270.6 | 16.9 | 0.17 | 3.38 | 0.63 | 4136.3 | 0.456 | 0.145 | 1.95 | 0.26 | -0.9 | -0.1 | -30.8 | 43.4 |
| SYZ | 96 | 64.8 | 26.3 | 5623.1 | 7.81 | 24.32 | 32.9 | 765.34 | 6706589 | 13103.8 | 15.77 | 0.21 | 4.09 | 0.50 | 4321.2 | 0.437 | 0.149 | 2.95 | 0.36 | -0.7 | -0.2 | -31.1 | 40.0 |
| SYZ | 477 | 46.4 | 26 | 4188.8 | 8.76 | 17.11 | 14.6 | 585.93 | 3180311 | 9600.5 | 12.97 | 0.12 | 4.26 | 0.57 | 5454.4 | 0.410 | 0.125 | 2.42 | 0.51 | -0.4 | -0.1 | -31.0 | 41.1 |
| NOR | 176 | 14.2 | 16.6 | 1331.7 | 9.96 | N.A | 62.3 | 157.96 | 10760437 | 71808.7 | 0.84 | 0.06 | 179.38 | 1.05 | 7191.3 | 0.421 | 0.143 | 13.69 | 0.31 | -1.0 | -0.1 | -30.3 | 48.2 |
| NOR | 157 | 14.6 | 18.2 | 544.5 | 3.84 | N.A | 23.0 | 167.00 | 6384002 | 76017.1 | 0.45 | 0.03 | 348.61 | 1.02 | 7187.4 | 0.398 | 0.127 | 17.01 | 0.34 | -1.2 | -0.3 | -30.5 | 46.7 |
| NOR | 415 | 15 | 14.8 | 1565.1 | 11.32 | N.A | 136.9 | 172.58 | 11947888 | 77995.9 | 0.79 | 0.06 | 198.63 | 1.04 | 7229.5 | 0.419 | 0.119 | 17.11 | 0.26 | -0.7 | -0.1 | -30.8 | 43.9 |
| NOR | 434 | 14.9 | 16.9 | 930.4 | 7.14 | N.A | 53.4 | 148.22 | 17548645 | 119566 | 0.50 | 0.06 | 393.49 | 1.08 | 4713.9 | 0.370 | 0.114 | 12.52 | 0.30 | -0.6 | -0.1 | -28.7 | 66.0 |

Species codes are as follows: *Alstonia scholaris* (ALS); *Elaeocarpus angustifolius* (ELA); *Argyrodendron peralatum* (ARG); *Castanospermum australe* (CAS); *Endiandra microneura* (END); *Myristica globosa* (MYR); *Syzygium graveolens* (SYZ); *Normanbya normanbyi* (NOR). Traits abbreviations: diameter at 1.3m height (cm): DBH; height (m): HT; maximum sap flow rate (cm^3^ hour^-1^): SF; maximum sap velocity (cm hour^-1^): SV; mean annual basal area increment (cm^2^ year^-1^): BA_i_; Huber value (unitless): HV; Sapwood area (cm^2^): SA; Theoretical specific conductivity (kg s^-1^ MPa^-1^): TSC; Vessel area (μm^2^): VA; Vessel density (*n* mm-²); VD; Vessel fraction: VF (unitless); leaf mass per area (g cm^-2^): Vulnerability index (unitless): VI; Wood density (g cm­^-3^):WD; leaf area (cm^2^): LA; leaf dry matter content (mg mg^-1^): LDMC; Leaf mass per unit area (mg mm^-2^): LMA; leaf slenderness: LS (unitless); minimum and maximum leaf water potentials (MPa): ψ_min_ and ψ_max_; Leaf carbon isotopes ratio (‰): δ^13^C; Water-use-efficiency (μmol mol^-1^): WUE_i_.
